# Elevated levels of plasma inactive stromal cell derived factor-1α predict poor long-term outcomes in diabetic patients following percutaneous coronary intervention

**DOI:** 10.1186/s12933-024-02197-z

**Published:** 2024-03-30

**Authors:** Yuichi Chikata, Hiroshi Iwata, Katsutoshi Miyosawa, Ryo Naito, Takuma Koike, Soshi Moriya, Hidetoshi Yasuda, Takehiro Funamizu, Shinichiro Doi, Hirohisa Endo, Hideki Wada, Manabu Ogita, Tomotaka Dohi, Takatoshi Kasai, Kikuo Isoda, Shinya Okazaki, Katsumi Miyauchi, Tohru Minamino

**Affiliations:** 1 Department of Cardiovascular Biology and Medicine, Juntendo University Graduate School of Medicine, 2-1-1 Hongo, Bunkyo-Ku, Tokyo, Japan; 2 Research Planning Department, Kowa Company, Ltd., Tokyo, Japan; 3 Department of Cardiology, Juntendo University Shizuoka Hospital, Shizuoka, Japan; 4 Department of Cardiology, Juntendo University Nerima Hospital, Tokyo, Japan

**Keywords:** Stromal cell derived factor-1α, Long-term outcome, PCI, Diabetes mellitus

## Abstract

**Background:**

Since the complication of diabetes mellitus (DM) is a risk for adverse cardiovascular outcomes in patients with coronary artery disease (CAD), appropriate risk estimation is needed in diabetic patients following percutaneous coronary intervention (PCI). However, there is no useful biomarker to predict outcomes in this population. Although stromal cell derived factor-1α (SDF-1α), a circulating chemokine, was shown to have cardioprotective roles, the prognostic impact of SDF-1α in diabetic patients with CAD is yet to be fully elucidated. Moreover, roles of SDF-1α isoforms in outcome prediction remain unclear. Therefore, this study aimed to assess the prognostic implication of three forms of SDF-1α including total, active, and inactive forms of SDF-1α in patients with DM and after PCI.

**Methods:**

This single-center retrospective analysis involved consecutive patients with diabetes who underwent PCI for the first time between 2008 and 2018 (n = 849). Primary and secondary outcome measures were all-cause death and the composite of cardiovascular death, non-fatal myocardial infarction, and ischemic stroke (3P-MACE), respectively. For determining plasma levels of SDF-1α, we measured not only total, but also the active type of SDF-1α by ELISA. Inactive isoform of the SDF-1α was calculated by subtracting the active isoform from total SDF-1α.

**Results:**

Unadjusted Kaplan–Meier analyses revealed increased risk of both all-cause death and 3P-MACE in patients with elevated levels of inactive SDF-1α. However, plasma levels of total and active SDF-1α were not associated with cumulative incidences of outcome measures. Multivariate Cox hazard analyses repeatedly indicated the 1 higher log-transformed inactive SDF-1α was significantly associated with increased risk of all-cause death (hazard ratio (HR): 2.64, 95% confidence interval (CI): 1.28–5.34, p = 0.008) and 3P-MACE (HR: 2.51, 95% CI: 1.12–5.46, p = 0.02). Moreover, the predictive performance of inactive SDF-1α was higher than that of total SDF-1α (C-statistics of inactive and total SDF-1α for all-cause death: 0.631 vs 0.554, for 3P-MACE: 0.623 vs 0.524, respectively).

**Conclusion:**

The study results indicate that elevated levels of plasma inactive SDF-1α might be a useful indicator of poor long-term outcomes in diabetic patients following PCI.

*Trial registration:* This study describes a retrospective analysis of a prospective registry database of patients who underwent PCI at Juntendo University Hospital, Tokyo, Japan (Juntendo Physicians’ Alliance for Clinical Trials, J-PACT), which is publicly registered (University Medical Information Network Japan—Clinical Trials Registry, UMIN-CTR 000035587).

**Supplementary Information:**

The online version contains supplementary material available at 10.1186/s12933-024-02197-z.

## Background

Type 2 diabetes mellitus (T2DM) is a global health problem due to its high worldwide prevalence, high economic cost of treatment, and associated atherosclerotic cardiovascular (CV) mortality and morbidity [1–3]. The clinical outcome of coronary artery disease (CAD) concomitant with T2DM following percutaneous coronary intervention (PCI) has been reported to be twofold to fourfold worse than those without T2DM [4–6]. Therefore, precise risk stratification for predicting long-term outcomes following PCI is particularly important in those who are complicated by diabetes. Previously, several biomarkers, such as lipoprotein (a), high-sensitivity C-reactive protein and 1,5-anhydroglucitol have been raised as candidates of outcome predictors following PCI [7–9]. However, very limited data are available regarding the usefulness of any biomarkers as prognostic indicators in diabetic patients with CAD.

The chemokine stromal cell derived factor-1α (SDF-1α/CXCL12) is a CXC chemokine with chemotactic effects on CXCR4/CXCR7 (CXCR: CXC chemokine receptor) expressing progenitor cells [10] and is known to play beneficial roles in cardiomyocyte repair and ventricular remodelling [11]. The prognostic value of circulating SDF-1α levels has been previously evaluated in several studies in patients with CAD [12–14], most of which reported that an elevated level of total SDF-1α was significantly associated with increased risk of poor outcomes in patients with CAD. Moreover, previous studies have suggested that SDF-1α play a key role in the pathogenesis of T2DM [15, 16]. However, no study specifically evaluated the prognostic implication of SDF-1α in diabetic patients with CAD. Moreover, these previous studies did not consider cleavage of SDF-1α by exopeptidases [17, 18] and they did not clarify any potential roles of isoforms, active or inactive isoforms, of SDF-1α in this patient population. Therefore, in the present study, we evaluated and compared the prognostic implications of three forms of SDF-1α, including total (conventionally measured plasma level of SDF-1α), active, and inactive forms of SDF-1α, in patients with T2DM following PCI.

## Methods

This study was conducted in accordance with the Declaration of Helsinki and was approved by the Institutional Review Board (IRB) of Juntendo University (IRB-ID: 20-287). The single-center prospective all-comer registry database of patients who underwent any type of PCI at Juntendo University Hospital, Tokyo, Japan (Juntendo Physicians’ Alliance for Clinical Trial, J-PACT) since 1984 is publicly registered (University Medical Information Network Japan—Clinical Trials Registry, UMIN-CTR 000035587). Written informed consent was obtained from all participants for the J-PACT registry which had no exclusion criteria as far as written informed consent was achieved.

### Participants, endpoints, follow-up, and follow-up duration

This study is a retrospective analysis of a portion of the J-PACT registry database involving consecutive 849 diabetic patients out of 4039 patients who underwent any type of PCI for CAD at Juntendo University Hospital between December 2008 and January 2018. Diabetes was defined as glycated hemoglobin (HbA1c-NG) ≥ 6.5% or if the patient was taking any diabetic medications at PCI procedure. Participants were divided into two groups according to the median of three forms of plasma SDF-1α levels (total SDF-1α: 2270 pg/mL, active SDF-1α: 686 pg/mL, inactive SDF-1α: 1537 pg/mL) at PCI procedure (high total, active, inactive SDF-1α group n = 425, low total, active, inactive SDF-1α group n = 424) and the incidence and risk of subsequent endpoints following PCI were assessed (Additional file 1: Fig. S1).

The primary and secondary outcome measures were all-cause death and the 3-point major adverse cardiovascular events (3P-MACE; a composite of CV death, non-fatal myocardial infarction and ischemic stroke), respectively. CV death was defined as a composite of the following types of death; sudden death in which non-cardiac death could not be excluded, and death due to myocardial infarction, heart failure, cardiogenic shock, a cerebrovascular event, or aortic diseases. In this prospective PCI registry database, patient follow-up was based on electronic chart review, as far as they were followed at Juntendo University Hospital. A prognosis survey questionnaire was mailed out every 5 years if they were transferred to other institutions. In cases in which no response was achieved, follow-up was terminated at the latest time point, at which their survival at our institution was confirmed, such as the last visit date to an outpatient clinic or the last day of any hospitalization. The median and range of the follow-up period were 4.1 and 0–10.4 years, respectively.

### Blood sampling and the measurement of plasma levels of total and active form of SDF-1α

Blood samples were obtained immediately prior to PCI through an inserted blood access (5 to 8 French in diameter) and stored at − 80 ℃ until measurement of total and active SDF-1α concentrations (n = 849). Plasma levels of both total and active SDF-1α were determined using an enzyme-linked immunosorbent assay (ELISA) according to a previous protocol [19]. We calculated the levels of inactive SDF-1α by subtracting the active isoform level from the total SDF-1α level.

### Statistical analysis

Quantitative variables are presented as the mean ± standard deviation or median with interquartile range (IQR) in accordance with the results of the Shapiro–Wilk normality test. Categorical variables are presented as the numbers and percentages. Quantitative data between groups were compared using Student’s *t*-test or the Wilcoxon rank sum test. Unadjusted Kaplan–Meier analysis evaluated the time to the cumulative incidence of endpoints followed by the log-rank test for comparisons. Univariate and multivariate analyses using the following models of Cox proportional hazards regression analyses (Models 1–3) calculated the hazard ratios (HRs) of total, active and inactive SDF-1α for all-cause death and 3P-MACE. Variables used in Models 1–3 were selected based on background demographics that were different between groups and associated with endpoints in univariate analyses. Moreover, the ratio of the number of the variables included in the occurrence of events for each model was set as approximately 1:10 [20, 21]. Model 1 included age (a continuous variable) and sex. Model 2 included age, sex, body mass index (BMI) (> 25), acute coronary syndrome (ACS), and chronic kidney disease (CKD) (≥ stage 3). Model 3 included age, sex, statins, multivessel disease, insulin, high-sensitivity C-reactive protein (hs-CRP) (a continuous variable), hemoglobin (a continuous variable), and B-type natriuretic peptide (BNP) (a continuous variable). Receiver operating characteristic (ROC) curves were obtained and the areas under the curve (C-statistic) were measured for all-cause death and 3P-MACE. We compared the two C-statistics. Furthermore, the net reclassification improvement (NRI) and the integrated discrimination improvement (IDI) were calculated to examine the additive effects of total, active and inactive SDF-1α levels on the predictive value of the baseline model. Statistical significance was defined as a p-value < 0.05 and analyses were performed using statistical software (JMP Pro 16.0; SAS Institute Inc., Cary, NC, USA and IBM SPSS Statistics, Version 26.0. Armonk, NY, USA).

## Results

### Superiority of inactive SDF-1α as an accurate prognostic indicator compared to total and active SDF-1α

**Fig. 1:**
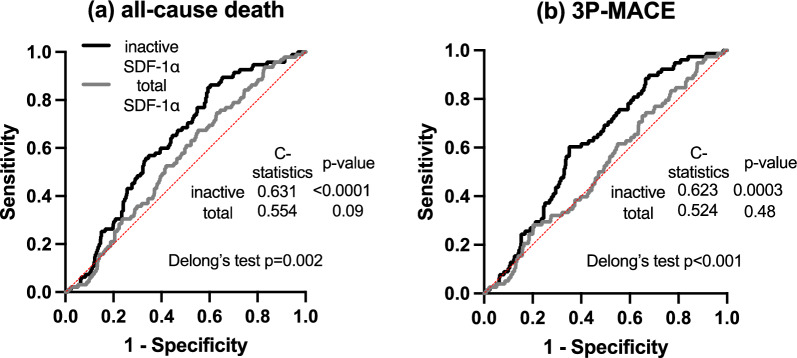
Predictive performance of inactive and total SDF-1α for all-cause death and 3P-MACE. Receiver operating characteristic (ROC) curves of inactive and total SDF-1α with reference line for all-cause death (**a**) and 3P-MACE (**b**)

The C-statistics of total and inactive SDF-1α for both endpoints were determined by ROC analysis. We found that the C-statistics of inactive SDF-1α for both endpoints were significantly higher than those of total SDF-1α (C-statistics of inactive and total SDF-1α for all-cause death: 0.631 vs 0.554, p = 0.002, for 3P-MACE: 0.623 vs 0.524, p < 0.001, respectively) (Fig. 1). Based on these findings, to assess the usefulness of each type of SDF-1α as a prognostic indicator, NRI and IDI were calculated by using 2 models in multivariate Cox proportional hazard analysis for all-cause death and 3P-MACE. Consequently, NRI and IDI for all-cause death were significantly improved by the addition of inactive SDF-1α to Model 1 and Model 2, while adding total and active SDF-1α did not improve them. Similar findings were obtained in the analyses for 3P-MACE (Table 1).

**Table 1:** Effects on the predictive performance by adding isoforms of SDF-1α on the baseline models

	NRI	p-value	IDI	p-value
*All-cause death*				
Model 1				
+ Total	0.11	0.33	0.0009	0.58
+ Active	0.15	0.30	0.003	0.28
+ Inactive	0.41	**0.0002**	0.01	**0.007**
Model 2				
+ Total	0.15	0.17	0.0002	0.85
+ Active	0.12	0.40	0.004	0.26
+ Inactive	0.29	**0.009**	0.007	**0.03**
*3P-MACE*				
Model 1				
+ Total	0.01	0.93	0.0002	0.72
+ Active	0.18	0.27	0.007	0.16
+ Inactive	0.45	**0.0001**	0.008	**0.01**
Model 2				
+ Total	− 0.08	1.48	0.0000	0.92
+ Active	0.16	0.33	0.01	0.12
+ Inactive	0.38	**0.001**	0.007	**0.01**

Model 1 included age and sex

Model 2 included age, sex, body mass index > 25, acute coronary syndrome and chronic kidney disease

### Baseline characteristics in 2 groups divided by the median of plasma inactive SDF-1α levels at PCI procedure

To clarify the similarities and differences in background demographics between patients with and without high inactive SDF-1α, the background demographics, comorbidities, and medications were compared. As shown in Table 2, most of the background demographics, including age, sex, left ventricular ejection fraction (LVEF), and number of diseased vessels were similar in both groups. Disease duration of diabetes was significantly longer in the high inactive SDF-1α group while HbA1c levels were similar in both groups. Hemoglobin levels and BMI were significantly lower in the high inactive SDF-1α group. Furthermore, in high inactive SDF-1α group, the proportions of patients with CKD and chronic hemodialysis were substantially higher, consistent with those having elevated serum creatinine and decreased estimated glomerular filtration rate (eGFR), compared to low inactive SDF-1α group. The proportion of patients with dyslipidemia and statin use was lower in the high inactive SDF-1α group. Additionally, the ratio of dipeptidyl peptidase-4 (DPP4) inhibitor use was significantly lower in the high inactive SDF-1α group, while there were no significant differences in other diabetic medications, including insulin.

**Table 2:** Baseline characteristics of study patients

	Overall	High inactive SDF-1α	Low inactive SDF-1α	p-value
	n = 849	n = 425	n = 424	
*Baseline characteristics*				
Age, years	67.8 ± 10.1	67.9 ± 10.7	67.7 ± 9.4	0.86
Male, n (%)	701 (82.6)	349 (82.1)	352 (83.0)	0.73
Body mass index, kg/m^2^	24.8 ± 3.8	24.4 ± 3.8	25.1 ± 3.7	**0.01**
Hypertension, n (%)	665 (78.3)	342 (80.5)	323 (76.2)	0.13
Dyslipidemia, n (%)	665 (78.3)	316 (74.4)	349 (82.3)	**0.005**
Current smoker, n (%)	206 (24.3)	97 (22.8)	109 (25.7)	0.33
Chronic kidney disease, n (%)	254 (29.9)	151 (35.5)	103 (24.3)	**0.0003**
Hemodialysis, n (%)	70 (8.2)	54 (12.7)	16 (3.8)	** < 0.0001**
Acute coronary syndrome, n (%)	193 (22.7)	92 (21.7)	101 (23.8)	0.45
LVEF, %	60.6 ± 12.7	59.6 ± 13.7	61.7 ± 11.5	0.06
Diabetes duration, years	14 (6, 21)	15 (7, 24)	13 (5, 20)	**0.04**
Number of diseased vessels	2.0 ± 0.8	2.0 ± 0.8	1.9 ± 0.8	0.46
*Vessel location*				
RCA, n (%)	253 (29.8)	126 (29.7)	127 (30.0)	0.92
LAD, n (%)	433 (51.0)	215 (50.6)	218 (51.4)	0.81
LCX, n (%)	165 (19.4)	86 (20.2)	79 (18.6)	0.56
*Laboratory findings*				
TC, mg/dL	166.4 ± 38.7	163.3 ± 40.7	169.6 ± 36.3	**0.02**
LDL-C, mg/dL (Friedewald)	95.8 ± 30.2	94.6 ± 31.7	97.0 ± 28.5	0.25
HDL-C, mg/dL	43.2 ± 13.2	42.1 ± 11.9	44.2 ± 14.3	**0.02**
TG, mg/dL	120 (88, 163)	113 (85, 158)	123 (91, 169)	**0.02**
FBG, mg/dL	133.4 ± 53.1	132.4 ± 55.3	134.5 ± 50.7	0.56
HbA1c, %	7.2 ± 1.1	7.2 ± 1.1	7.2 ± 1.0	0.57
hs-CRP, mg/L	0.09 (0.03, 0.28)	0.09 (0.03, 0.33)	0.08 (0.04, 0.22)	0.49
Hemoglobin, g/dL	13.3 ± 1.9	13.1 ± 2.0	13.5 ± 1.8	**0.009**
Creatinine, mg/dL	0.80 (0.67, 0.98)	0.81 (0.68, 1.06)	0.79 (0.67, 0.93)	**0.01**
eGFR, ml/min/1.73m^2^	69.4 ± 28.2	66.4 ± 32.1	72.4 ± 23.4	**0.002**
BNP, pg/mL	48.7 (21.3, 126.5)	63.3 (25.2, 173.3)	40.3 (18.7, 90.5)	** < 0.0001**
*Medication*				
Sulfonylurea, n (%)	228 (26.9)	123 (28.9)	105 (24.8)	0.18
Metformin, n (%)	161 (19.0)	79 (18.6)	82 (19.4)	0.77
Thiazolidinedione, n (%)	94 (11.1)	40 (9.4)	54 (12.8)	0.12
SGLT-2 inhibitor, n (%)	17 (2.0)	9 (2.1)	8 (1.9)	0.81
DPP4 inhibitor, n (%)	310 (36.6)	137 (32.2)	173 (40.9)	**0.009**
GLP-1 receptor agonist, n (%)	6 (0.7)	2 (0.5)	4 (1.0)	0.41
α-Glucosidase inhibitor, n (%)	182 (21.5)	92 (21.7)	90 (21.3)	0.90
Glinide, n (%)	71 (8.4)	31 (7.3)	40 (9.5)	0.26
Insulin, n (%)	213 (25.1)	117 (27.5)	96 (22.7)	0.10
ACE-I/ ARB, n (%)	468 (55.1)	232 (54.6)	236 (55.7)	0.75
β-Blocker, n (%)	391 (46.1)	191 (44.9)	200 (47.2)	0.51
Statin, n (%)	581 (68.6)	272 (64.0)	309 (73.2)	**0.004**
Ezetimibe, n (%)	50 (5.9)	22 (5.2)	28 (6.6)	0.37
Fibrate, n (%)	34 (4.0)	17 (4.0)	17 (4.0)	0.99

*LVEF* left ventricular ejection fraction, *RCA* right coronary artery, *LAD* left anterior descending artery, *LCX* left circumflex artery, *TC* total cholesterol, *LDL-C* low-density lipoprotein, *HDL-C* high-density lipoprotein, *TG* triglycerides, *FBG* fasting blood glucose, *HbA1c* glycated hemoglobin, *hs-CRP* high-sensitivity C-reactive protein, *eGFR* estimated glomerular filtration rate, *BNP* B-type natriuretic peptide, *SGLT-2* sodium-glucose co-transporter-2, *DPP4* Dipeptidyl peptidase-4, *GLP-1* glucagon-like peptide-1, *ACE-I* angiotensin-converting enzyme inhibitors, *ARB* angiotensin receptor blockers

### Prognostic impact of inactive SDF-1α in diabetic patients after PCI

**Fig. 2:**
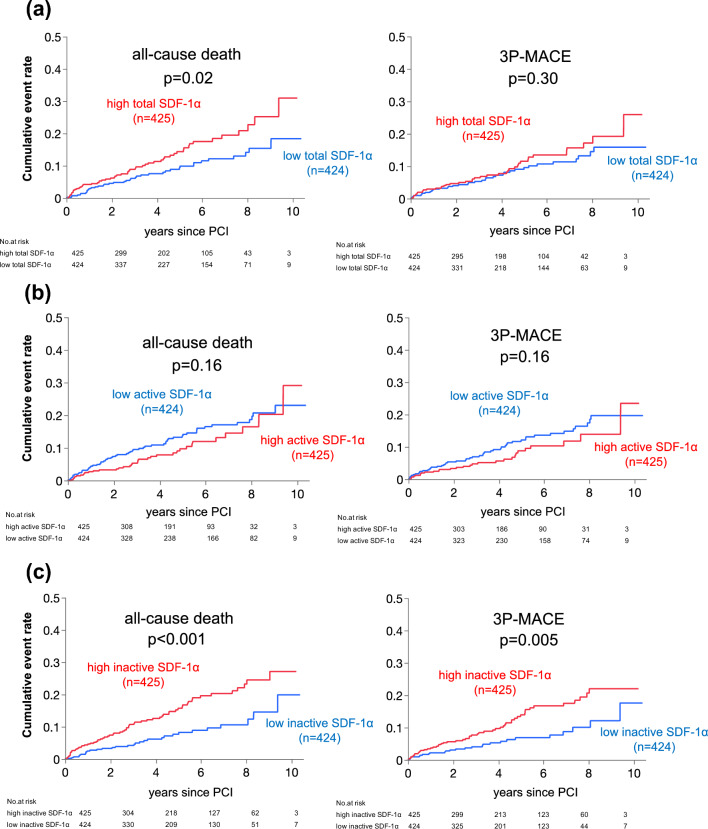
Cumulative incidences of adverse events in groups divided by median levels of total, active and inactive plasma SDF-1α. **a** Cumulative incidences of all-cause death and 3P-MACE in patients with high and low plasma total SDF-1α levels. **b** Cumulative incidences of all-cause death and 3P-MACE in patients with high and low plasma active SDF-1α levels. **c** Cumulative incidences of all-cause death and 3P-MACE in patients with high and low plasma inactive SDF-1α levels

The median and range of the follow-up period were 4.1 and 0–10.4 years, respectively. During the follow-up period following PCI, the total numbers of all-cause death and 3P-MACE were 95 (11.2%) and 78 (9.2%) out of 849 participants, respectively. Unadjusted Kaplan–Meier analysis followed by the log-rank comparison test demonstrated that the cumulative incidences of both all-cause death and 3P-MACE were significantly higher in the high inactive SDF-1α group compared to the low inactive SDF-1α group (Fig. 2c). Moreover, a similar result was observed in the cumulative incidence of all-cause death between the high and low total SDF-1α groups, however, there was no difference in that of 3P-MACE (Fig. 2a). In contrast, the cumulative incidences of both endpoints were slightly lower in the high active SDF-1α group compared to the low active SDF-1α group (Fig. 2b).

**Fig. 3:**
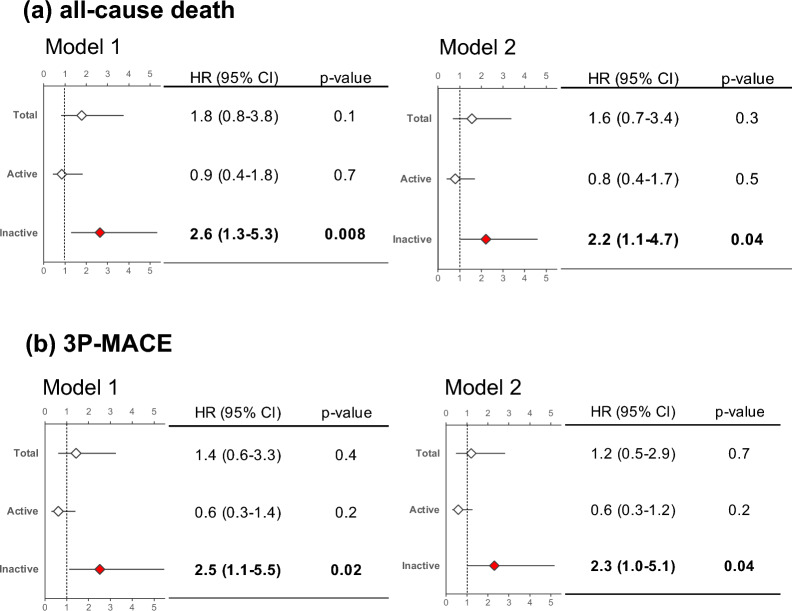
Prognostic impact of each isoform of SDF-1α in 2 Cox proportional hazard models. Hazard ratios were calculated by multivariate Cox proportional hazard analyses by using 2 different models. Model 1: age and sex, Model 2: included age, sex, body mass index (BMI) (> 25), acute coronary syndrome (ACS), and chronic kidney disease (CKD) (≥ stage 3) *HR* hazard ratio, *95%CI* confidence interval

Univariate Cox proportional hazard analyses for calculating hazard ratios for subsequent all-cause death and 3P-MACE following PCI revealed that an elevated level of plasma inactive SDF-1α at procedure was significantly associated with increased risk of both endpoints (Additional file 1: Tables S1 and S2). Multivariate Cox proportional analyses using 3 models in which covariates were selected based on background demographics and univariate analyses (Additional file 1: Tables S1 and S2) indicated that elevated levels of plasma inactive SDF-1α were significantly associated with increased risk of all-cause death and 3P-MACE (Fig. 3 and Additional file 1: Table S3). However, elevated levels of plasma total SDF-1α were not significantly associated with increased risk of both endpoints. Consistent with the Kaplan–Meier analysis, elevated levels of plasma active SDF-1α tended to be associated with decreased risk of both endpoints (Fig. 3 and Additional file 1: Table S3).

## Discussion

This single-center observational study of a prospective PCI registry database evaluated and compared the prognostic implications of three forms of SDF-1α (total, active, and inactive) in high CV risk patients for the first time. The cumulative incidences and risk for all-cause death and 3P-MACE were assessed in two patient groups divided according to the median of the plasma levels of each isoform of SDF-1α at PCI procedure. The primary findings are as follows. (1) Predictive performance of inactive SDF-1α for all-cause death and 3P-MACE was higher compared to those of total SDF-1α. Additionally, the predictability of all-cause death and 3P-MACE was significantly improved when inactive SDF-1α was added to the Cox proportional hazard models compared to total and active SDF-1α. (2) Unadjusted Kaplan–Meier analyses showed the higher cumulative incidences of both endpoints in patients with high inactive SDF-1α. (3) Multivariate Cox proportional hazard analyses using 3 models continuously showed that an elevated plasma inactive SDF-1α level was significantly associated with increased risk of all-cause death and 3P-MACE.

SDF-1α, also referred to as C-X-C motif chemokine 12 (CXCL12), is a small peptide consisting of 89 amino acids, which is originally produced at the platelet membrane, and plays a prominent role in hematopoiesis, angiogenesis, immunogenesis, stem cell mobilization, and tissue repair through its receptors CXCR4 and CXCR7 [10, 22]. Previous studies have reported that SDF-1α and its receptor, CXCR4, or SDF-1/CXCR4 axis was cardioprotective after myocardial infarction, including attenuation of adverse ventricular remodelling and preservation of ventricular function [23, 24]. SDF-1 has emerged as one of the key regulators in cardiac tissue repair [25], which have been shown to induce mobilization of bone marrow-derived and cardiac premature cells to the injured cardiac tissues, promote angiogenesis, inhibit cardiac myocyte cell apoptosis, and finally lead to the inhibition of cardiac remodelling [26, 27]. Clinically, a placebo-controlled trial evaluated the safety and efficacy of plasmid-induced SDF-1 gene overexpression, which was delivered via endomyocardial injection into patients with ischemic heart failure. The study described the potential for attenuating LV remodelling and improving LVEF in high-risk ischemic cardiomyopathy [28]. Furthermore, several experimental studies have indicated that SDF-1α/CXCR4 axis has both beneficial and detrimental effects on glycometabolism. It has been reported that the SDF-1α/CXCR4 axis improve glycemic control by inhibiting dedifferentiation of islet β cells, but exacerbate it by reducing insulin effectiveness on adipocytes [15, 16]. Therefore, the overall association between the progression of diabetes and circulating total SDF-1α level remains inconclusive.

Myocardial ischemia results in an elevated plasma level of SDF-1α, which was reported to associate with increased risk of heart failure and all-cause mortality in patients with CAD [29]. When cardiomyocytes are under ischemic conditions, hypoxia inducible factor 1α (HIF-1α) activates the SDF-1α/CXCR4 pathway [30, 31]. While the functional roles of the CXCR7 pathway have yet to be clarified, the SDF-1α/CXCR4 pathway has been intensively studied and found to exert cardiovascular protective effects by Erk and Akt activation [32]. Moreover, SDF-1α/CXCR4 signalling promotes angiogenesis in ischemic tissues by enhancing local angiogenic mechanisms and by the recruitment or mobilization of endothelial progenitor cells (EPCs) [17, 23, 33]. Additionally, accumulating evidence has suggested that SDF-1α plays a role in ischemic preconditioning of the cardiac tissue, which is known to be associated with the reduction of infarct size in patients with myocardial infarction [34] and with better clinical outcomes in patients following PCI and cardiac surgery [19, 35–37].

SDF-1α is biologically active only before the cleavage (active SDF1α) by various exopeptidases, such as DPP4 and the family of matrix metalloproteinases (MMP) [17, 18]. After shedding amino acids from the active form, SDF-1α (inactive SDF1α) was suggested not only to lose its chemotactic functions, but also to be a potential antagonist of CXCR4 pathways [18]. The nonfunctional or even potential antagonistic properties of inactive SDF-1α may interfere with or attenuate the beneficial cardioprotective properties of SDF-1α.

Previous studies assessing circulating SDF-1α levels as a prognostic indicator in CAD patients have shown that its elevation was associated with increased risk of adverse cardiovascular events, including myocardial infarction and CV mortality in chronic coronary syndrome patients and ACS patients [12–14]. Conversely, other studies have reported a reduction in circulating SDF-1α in patients with myocardial infarction [38], and with stable and unstable angina compared to healthy controls [39]. As all these studies utilized ELISA antibodies recognizing SDF-1α without considering cleavage, the conjugated plasma levels of both active plus inactive SDF-1α (total SDF-1α) were measured, which might be responsible for the inconsistency regarding the change in ischemic heart disease and the prognostic impact of plasma SDF-1α. Therefore, the separate measurement of these two isoforms of SDF-1α might be very helpful for the clarification of its biological roles. Moreover, it allows us to assess the usefulness of SDF-1α as a biomarker to predict outcomes in patients with ischemic heart disease. In the present study, we found that elevation of inactive SDF-1α which was cleaved from the active form was more closely associated with increased risk of all-cause mortality and 3P-MACE compared to the active form and total SDF-1α. Based on these novel findings in this study, we can speculate that disturbance of the beneficial effects of the SDF-1α/CXCR4 pathway by the inactivated form of SDF-1α itself may lead to adverse outcomes in diabetic patients with CAD. Accordingly, an elevated level of inactive SDF-1α may be a more accurate prognostic indicator compared to the active form or total SDF-1α, in diabetic patients following PCI.

### Limitations

There are several limitations to this study. First, it was a single center, retrospective observational study involving almost solely Japanese patients and therefore did not compare findings amongst different ethnicities. Further large-scale clinical studies are warranted to confirm our results. Second, the possibility that unknown confounders might have had an influence on the results could not be completely eliminated, even though multivariate analyses adjusted for baseline characteristics and known prognostic factors. Third, plasma levels of inactive SDF-1α were not measured directly but rather were estimated by the differences between total SDF-1α levels and active SDF-1α levels. Fourth, approximately 30% of participants were prescribed DPP4 inhibitors. As DPP4 is one of the exopeptidases which cleave SDF-1α, the potential effects of taking DPP4 inhibitors on the prognostic implication of SDF-1α may need to be considered, although the findings of our sub-analysis with DPP4 inhibitors (36% of study participants) were very similar to those for total participants and without DPP4 inhibitors (data not shown). Fifth, previous studies suggested possible associations between the SDF-1α/CXCR4 axis and development of various clinical conditions such as immunological diseases and cancers [40, 41]. However, as this study did not assess the immunological diseases and cancers, their potential influence on the patient outcomes cannot be completely excluded. Despite these major limitations, this study is strengthened by providing novel evidence regarding the clinical utility of inactive SDF-1α.

## Conclusions

The results of this retrospective observational study indicate that elevated plasma levels of inactive SDF-1α were significantly associated with poor long-term outcomes in diabetic patients following PCI. Moreover, the predictive performance of inactive SDF-1α was more accurate than that of conventionally used plasma total SDF-1α. Preprocedural plasma inactive SDF-1α levels may be a useful prognostic indicator in diabetic patients following PCI.

### Supplementary Information

**Additional file 1: Table S1.** Univariate Cox proportional hazard analyses for predictors of all-cause death following PCI. **Table S2.** Univariate Cox proportional hazard analyses for predictors of 3P-MACE following PCI. **Table S3.** Multivariate cox proportional hazard analyses using 3 models to assess the hazard ratios of total/active/inactive SDF-1α for all-cause death and 3P-MACE. **Figure S1.** Flow diagram of the study participants.

## Data Availability

The datasets used and/or analyzed during the current study are available from the corresponding author on reasonable request.

## Abbreviations

DM: Diabetes mellitus
CAD: Coronary artery disease
PCI: Percutaneous coronary intervention
SDF-1α: Stromal cell-derived factor-1α
3P-MACE: 3-Point major adverse cardiovascular events
ELISA: Enzyme-linked immunosorbent assay
HRs: Hazard ratios
CI: Confidence interval
T2DM: Type 2 diabetes mellitus
CV: Cardiovascular
CXCR: CXC chemokine receptor
IRB: Institutional Review Board
HbA1c: Glycated hemoglobin
IQR: Interquartile range
BMI: Body mass index
ACS: Acute coronary syndrome
CKD: Chronic kidney disease
NRI: Net reclassification improvement
IDI: Integrated discrimination improvement
LVEF: Left ventricular ejection fraction
eGFR: Estimated glomerular filtration rate
CXCL12: C-X-C motif chemokine
HIF1α: Hypoxia inducible factor 1α
EPCs: Endothelial progenitor cells
MMP: Matrix metalloproteinases
