# Supplementary material for: Elevated levels of plasma inactive stromal cell derived factor-1α predict poor long-term outcomes in diabetic patients following percutaneous coronary intervention
Source: Cardiovasc Diabetol. 2024 Mar 30;23:114. doi: 10.1186/s12933-024-02197-z (PMC10981820; doi:10.1186/s12933-024-02197-z)

**ADDITIONAL MATERIALS**

**Table S1**

Univariate Cox proportional hazard analyses for predictors of all-cause death following PCI

|  | **HR** | **95% CI** | **p-value** |
| --- | --- | --- | --- |
| **Age (1 year older)** | **1.07** | **1.05–1.10** | **<0.0001** |
| **Male** | **2.21** | **1.18–4.73** | **0.01** |
| **BMI >25** | **0.38** | **0.23–0.61** | **<0.0001** |
| Hypertension | 0.87 | 0.54–1.47 | 0.60 |
| Dyslipidemia | 1.04 | 0.64–1.77 | 0.87 |
| Acute coronary syndrome | 1.00 | 0.61–1.58 | 0.99 |
| **Multivessel disease** | **1.88** | **1.17–3.17** | **0.008** |
| **Chronic kidney disease** | **2.23** | **1.48–3.34** | **0.0001** |
| **LVEF^2^ (1% higher)** | **0.96** | **0.94–0.97** | **<0.0001** |
| Former smoker | 1.40 | 0.93–2.09 | 0.10 |
| Beta-blockers | 0.78 | 0.52–1.17 | 0.23 |
| ACEIs^3^/ARBs^4^ | 1.01 | 0.68–1.53 | 0.95 |
| **Statins** | **0.59** | **0.40–0.89** | **0.01** |
| Ezetimibe | 0.58 | 0.14–1.55 | 0.31 |
| **Insulin** | **2.36** | **1.56–3.55** | **<0.0001** |
| **Hemoglobin (1 g/dL higher)** | **0.68** | **0.61–0.77** | **<0.0001** |
| **Triglycerides (1 mg/dL higher)** | **0.99** | **0.990–0.998** | **0.0006** |
| LDL-C^5^ (1 mg/dL higher) | 0.99 | 0.99–1.00 | 0.14 |
| HDL-C^6^ (1 mg/dL higher) | 0.99 | 0.97–1.00 | 0.18 |
| HbA1c-NG^7^ (1 % higher) | 0.85 | 0.68–1.04 | 0.11 |
| Fasting blood glucose (1 mg/dL higher) | 1.00 | 0.99–1.01 | 0.27 |
| **Log BNP^8^ (1 higher)** | **3.97** | **2.82–5.56** | **<0.0001** |
| **eGFR^9^ (10 mL/min/1.73m^2^ higher)** | **0.81** | **0.76–0.88** | **<0.0001** |
| **hs-CRP^10^ (0.1 mg/dL higher)** | **1.03** | **1.02–1.04** | **<0.0001** |
| Log total SDF-1α^11^ (1 higher) | 1.83 | 0.86–3.80 | 0.11 |
| Log active SDF-1α^11^ (1 higher) | 1.03 | 0.51–2.19 | 0.95 |
| **Log inactive SDF-1α^11^ (1 higher)** | **2.59** | **1.24–5.23** | **0.01** |
| 1 body mass index, 2 left ventricular ejection fraction, 3 angiotensin converting enzyme inhibitors, 4 angiotensin Ⅱ receptor blockers, 5 low-density lipoprotein, 6 high-density lipoprotein, 7 glycated hemoglobin, 8 B-type natriuretic peptide, 9 estimated glomerular filtration rate, 10 high-sensitivity C-reactive protein, 11 stromal cell derived factor-1α | | | |

**Table S2**

Univariate Cox proportional hazard analyses for predictors of 3P-MACE following PCI

|  | **HR** | **95% CI** | **p-value** |
| --- | --- | --- | --- |
| **Age (1 year older)** | **1.03** | **1.01–1.06** | **0.007** |
| Male | 1.42 | 0.75–2.69 | 0.282 |
| BMI >25 | 0.90 | 0.57–1.42 | 0.649 |
| Hypertension | 0.99 | 0.56–1.73 | 0.959 |
| Dyslipidemia | 1.09 | 0.62–1.92 | 0.762 |
| Acute coronary syndrome | 1.25 | 0.76–2.05 | 0.370 |
| Multivessel disease | 1.66 | 0.97–2.81 | 0.063 |
| **Chronic kidney disease** | **2.25** | **1.44–3.51** | **0.0004** |
| **LVEF^2^ (1% higher)** | **0.97** | **0.96–0.99** | **0.005** |
| Former smoker | 1.43 | 0.92–2.24 | 0.112 |
| Beta-blockers | 1.02 | 0.65–1.59 | 0.936 |
| ACEIs^3^/ARBs^4^ | 1.00 | 0.64–1.57 | 0.99 |
| **Statins** | **0.58** | **0.37–0.91** | **0.018** |
| Ezetimibe | 0.71 | 0.22–2.25 | 0.559 |
| **Insulin** | **2.27** | **1.44–3.58** | **0.0004** |
| **Hemoglobin (1 g/dL higher)** | **0.70** | **0.61–0.80** | **<0.0001** |
| Triglycerides (1 mg/dL higher) | 0.99 | 0.994–1.001 | 0.174 |
| LDL-C^5^ (1 mg/dL higher) | 0.99 | 0.992–1.007 | 0.90 |
| HDL-C^6^ (1 mg/dL higher) | 0.99 | 0.980–1.014 | 0.83 |
| HbA1c-NG^7^ (1 % higher) | 1.11 | 0.92–1.32 | 0.248 |
| Fasting blood glucose (1 mg/dL higher) | 1.00 | 0.999–1.007 | 0.062 |
| **Log BNP^8^ (1 higher)** | **3.54** | **2.42–5.14** | **<0.0001** |
| **eGFR^9^ (10 mL/min/1.73m^2^ higher)** | **0.80** | **0.74–0.87** | **<0.0001** |
| **hs-CRP^10^ (0.1 mg/dL higher)** | **1.02** | **1.01–1.03** | **<0.0001** |
| Log total SDF-1α^11^ (1 higher) | 1.42 | 0.61–3.20 | 0.410 |
| Log active SDF-1α^11^ (1 higher) | 0.67 | 0.32–1.49 | 0.307 |
| **Log inactive SDF-1α^11^ (1 higher)** | **2.44** | **1.09–5.30** | **0.027** |
| 1 body mass index, 2 left ventricular ejection fraction, 3 angiotensin converting enzyme inhibitors, 4 angiotensin Ⅱ receptor blockers, 5 low-density lipoprotein, 6 high-density lipoprotein, 7 glycated hemoglobin, 8 B-type natriuretic peptide, 9 estimated glomerular filtration rate, 10 high-sensitivity C-reactive protein, 11 stromal cell derived factor-1α | | | |

**Table S3**

Multivariate cox proportional hazard analyses using 3 models to assess the hazard ratios of total/active/inactive SDF-1α for all-cause death and 3P-MACE

1. **total SDF-1α**

**Model 1** **for all-cause death**

|  | **HR** | **95% CI** | **p-value** |
| --- | --- | --- | --- |
| **Age (1 year older)** | **1.08** | **1.05–1.10** | **<0.0001** |
| **Male** | **2.74** | **1.38–5.46** | **0.004** |
| Log total SDF-1α (1 higher) | 1.78 | 0.82–3.75 | 0.13 |

**Model 2** **for all-cause death**

|  | **HR** | **95% CI** | **p-value** |
| --- | --- | --- | --- |
| **Age (1 year older)** | **1.06** | **1.04–1.09** | **<0.0001** |
| **Male** | **2.70** | **1.35–5.39** | **0.005** |
| **BMI >25** | **0.50** | **0.30–0.82** | **0.006** |
| Acute coronary syndrome | 0.98 | 0.60–1.60 | 0.94 |
| **Chronic kidney disease** | **1.72** | **1.13–2.63** | **0.01** |
| Log total SDF-1α (1 higher) | 1.56 | 0.69–3.39 | 0.27 |

**Model 3 for all-cause death**

|  | **HR** | **95% CI** | **p-value** |
| --- | --- | --- | --- |
| **Age (1 year older)** | **1.06** | **1.02–1.10** | **0.001** |
| **Male** | **11.41** | **1.55–84.04** | **0.02** |
| Statins | 0.55 | 0.29–1.05 | 0.07 |
| Multivessel disease | 1.82 | 0.83–3.98 | 0.13 |
| **Insulin** | **2.20** | **1.15–4.22** | **0.02** |
| **hs-CRP (0.1 mg/dL higher)** | **1.02** | **1.01–1.04** | **0.0006** |
| Hemoglobin (1 g/dL higher) | 0.83 | 0.70–1.00 | 0.051 |
| **Log BNP (1 higher)** | **2.76** | **1.66–4.61** | **<0.0001** |
| Log total SDF-1α (1 higher) | 1.66 | 0.46–5.99 | 0.44 |

**Model 1** **for 3P-MACE**

|  | **HR** | **95% CI** | **p-value** |
| --- | --- | --- | --- |
| **Age (1 year older)** | **1.04** | **1.01–1.06** | **0.004** |
| Male | 1.58 | 0.83–3.01 | 0.16 |
| Log total SDF-1α (1 higher) | 1.43 | 0.62–3.32 | 0.40 |

**Model 2** **for 3P-MACE**

|  | **HR** | **95% CI** | **p-value** |
| --- | --- | --- | --- |
| **Age (1 year older)** | **1.03** | **1.00–1.05** | **0.04** |
| Male | 1.64 | 0.86–3.11 | 0.13 |
| BMI >25 | 1.04 | 0.65–1.66 | 0.87 |
| Acute coronary syndrome | 1.31 | 0.79–2.17 | 0.30 |
| **Chronic kidney disease** | **2.03** | **1.27–3.23** | **0.003** |
| Log total SDF-1α (1 higher) | 1.20 | 0.50–2.88 | 0.68 |

**Model 3 for 3P-MACE**

|  | **HR** | **95% CI** | **p-value** |
| --- | --- | --- | --- |
| Age (1 year older) | 1.04 | 1.00–1.08 | 0.08 |
| Male | 3.82 | 0.89–16.42 | 0.07 |
| Statins | 0.75 | 0.34–1.67 | 0.48 |
| Multivessel disease | 1.81 | 0.72–4.60 | 0.21 |
| **Insulin** | **2.26** | **1.04–4.91** | **0.04** |
| **hs-CRP (0.1 mg/dL higher)** | **1.03** | **1.01–1.04** | **0.009** |
| Hemoglobin (1 g/dL higher) | 0.82 | 0.66–1.02 | 0.07 |
| **Log BNP (1 higher)** | **2.22** | **1.18–4.19** | **0.01** |
| Log total SDF-1α (1 higher) | 3.04 | 0.74–12.59 | 0.12 |

1. **active SDF-1α**

**Model 1** **for all-cause death**

|  | **HR** | **95% CI** | **p-value** |
| --- | --- | --- | --- |
| **Age (1 year older)** | **1.08** | **1.05–1.11** | **<0.0001** |
| Male | 1.81 | 0.84–3.90 | 0.13 |
| Log active SDF-1α (1 higher) | 0.85 | 0.42–1.83 | 0.66 |

**Model 2** **for all-cause death**

|  | **HR** | **95% CI** | **p-value** |
| --- | --- | --- | --- |
| **Age (1 year older)** | **1.06** | **1.02–1.09** | **0.001** |
| Male | 1.78 | 0.82–3.85 | 0.14 |
| **BMI >25** | **0.43** | **0.22–0.84** | **0.01** |
| Acute coronary syndrome | 1.26 | 0.71–2.23 | 0.43 |
| **Chronic kidney disease** | **1.92** | **1.07–3.42** | **0.03** |
| Log active SDF-1α (1 higher) | 0.80 | 0.40–1.70 | 0.54 |

**Model 3 for all-cause death**

|  | **HR** | **95% CI** | **p-value** |
| --- | --- | --- | --- |
| **Age (1 year older)** | **1.06** | **1.01–1.11** | **0.02** |
| Male | 5.26 | 0.69–40.06 | 0.11 |
| Statins | 0.44 | 0.18–1.03 | 0.06 |
| Multivessel disease | 1.80 | 0.65–5.00 | 0.26 |
| Insulin | 2.26 | 0.98–5.25 | 0.06 |
| hs-CRP (0.1 mg/dL higher) | 1.02 | 0.99–1.04 | 0.11 |
| Hemoglobin (1 g/dL higher) | 0.88 | 0.69–1.14 | 0.31 |
| **Log BNP (1 higher)** | **3.10** | **1.61–6.25** | **0.001** |
| Log active SDF-1α (1 higher) | 0.67 | 0.23–2.22 | 0.49 |

**Model 1** **for 3P-MACE**

|  | **HR** | **95% CI** | **p-value** |
| --- | --- | --- | --- |
| Age (1 year older) | 1.03 | 0.99–1.06 | 0.10 |
| Male | 1.09 | 0.50–2.40 | 0.83 |
| Log active SDF-1α (1 higher) | 0.62 | 0.30–1.40 | 0.23 |

**Model 2** **for 3P-MACE**

|  | **HR** | **95% CI** | **p-value** |
| --- | --- | --- | --- |
| Age (1 year older) | 1.01 | 0.98–1.05 | 0.45 |
| Male | 1.08 | 0.49–2.39 | 0.84 |
| BMI >25 | 0.92 | 0.48–1.75 | 0.79 |
| **Acute coronary syndrome** | **1.86** | **1.00–3.44** | **0.048** |
| **Chronic kidney disease** | **2.44** | **1.28–4.65** | **0.007** |
| Log active SDF-1α (1 higher) | 0.59 | 0.29–1.22 | 0.16 |

**Model 3 for 3P-MACE**

|  | **HR** | **95% CI** | **p-value** |
| --- | --- | --- | --- |
| Age (1 year older) | 1.01 | 0.97–1.06 | 0.70 |
| Male | 3.77 | 0.48–29.50 | 0.21 |
| Statins | 1.04 | 0.30–3.59 | 0.95 |
| Multivessel disease | 1.34 | 0.41–4.32 | 0.63 |
| **Insulin** | **2.95** | **1.08–8.12** | **0.04** |
| **hs-CRP (0.1 mg/dL higher)** | **1.04** | **1.01–1.06** | **0.004** |
| **Hemoglobin (1 g/dL higher)** | **0.75** | **0.57–1.01** | **0.048** |
| Log BNP (1 higher) | 1.69 | 0.79–3.69 | 0.18 |
| Log active SDF-1α (1 higher) | 1.05 | 0.31–4.70 | 0.95 |

1. **inactive SDF-1α**

**Model 1** **for all-cause death**

|  | **HR** | **95% CI** | **p-value** |
| --- | --- | --- | --- |
| **Age (1 year older)** | **1.08** | **1.05–1.10** | **<0.0001** |
| **Male** | **2.64** | **1.32–5.27** | **0.006** |
| **Log inactive SDF-1α (1 higher)** | **2.64** | **1.28–5.34** | **0.008** |

**Model 2** **for all-cause death**

|  | **HR** | **95% CI** | **p-value** |
| --- | --- | --- | --- |
| **Age (1 year older)** | **1.06** | **1.04–1.09** | **<0.0001** |
| **Male** | **2.62** | **1.31–5.23** | **0.006** |
| **BMI >25** | **0.52** | **0.31–0.85** | **0.009** |
| Acute coronary syndrome | 1.05 | 0.66–1.69 | 0.83 |
| **Chronic kidney disease** | **1.65** | **1.08–2.53** | **0.02** |
| **Log inactive SDF-1α (1 higher)** | **2.21** | **1.05–4.67** | **0.04** |

**Model 3 for all-cause death**

|  | **HR** | **95% CI** | **p-value** |
| --- | --- | --- | --- |
| **Age (1 year older)** | **1.07** | **1.03–1.11** | **0.0003** |
| **Male** | **10.67** | **1.45–78.52** | **0.02** |
| Statins | 0.55 | 0.29–1.03 | 0.06 |
| Multivessel disease | 1.66 | 0.77–3.59 | 0.20 |
| Insulin | 1.93 | 0.998–3.72 | 0.051 |
| **hs-CRP (0.1 mg/dL higher)** | **1.03** | **1.01–1.04** | **0.0002** |
| Hemoglobin (1 g/dL higher) | 0.84 | 0.70–1.01 | 0.07 |
| **Log BNP (1 higher)** | **2.77** | **1.64–4.71** | **0.0002** |
| **Log inactive SDF-1α (1 higher)** | **4.39** | **1.40–13.79** | **0.01** |

**Model 1** **for 3P-MACE**

|  | **HR** | **95% CI** | **p-value** |
| --- | --- | --- | --- |
| **Age (1 year older)** | **1.04** | **1.01–1.06** | **0.004** |
| Male | 1.54 | 0.81–2.92 | 0.19 |
| **Log inactive SDF-1α (1 higher)** | **2.51** | **1.12–5.46** | **0.02** |

**Model 2** **for 3P-MACE**

|  | **HR** | **95% CI** | **p-value** |
| --- | --- | --- | --- |
| **Age (1 year older)** | **1.03** | **1.00–1.05** | **0.03** |
| Male | 1.60 | 0.84–3.05 | 0.15 |
| BMI >25 | 1.09 | 0.68–1.74 | 0.72 |
| Acute coronary syndrome | 1.37 | 0.84–2.24 | 0.21 |
| **Chronic kidney disease** | **1.92** | **1.20–3.07** | **0.006** |
| **Log inactive SDF-1α (1 higher)** | **2.30** | **1.01–5.09** | **0.04** |

**Model 3 for 3P-MACE**

|  | **HR** | **95% CI** | **p-value** |
| --- | --- | --- | --- |
| **Age (1 year older)** | **1.04** | **1.00–1.09** | **0.045** |
| Male | 3.62 | 0.84–15.65 | 0.09 |
| Statins | 0.75 | 0.34–1.65 | 0.47 |
| Multivessel disease | 1.65 | 0.66–4.17 | 0.29 |
| Insulin | 1.99 | 0.91–4.35 | 0.09 |
| **hs-CRP (0.1 mg/dL higher)** | **1.03** | **1.01–1.05** | **0.006** |
| Hemoglobin (1 g/dL higher) | 0.82 | 0.66–1.02 | 0.08 |
| **Log BNP (1 higher)** | **2.28** | **1.17–4.46** | **0.02** |
| **Log inactive SDF-1α (1 higher)** | **5.58** | **1.50–20.70** | **0.01** |

**Figure S1: Flow diagram of the study participants**


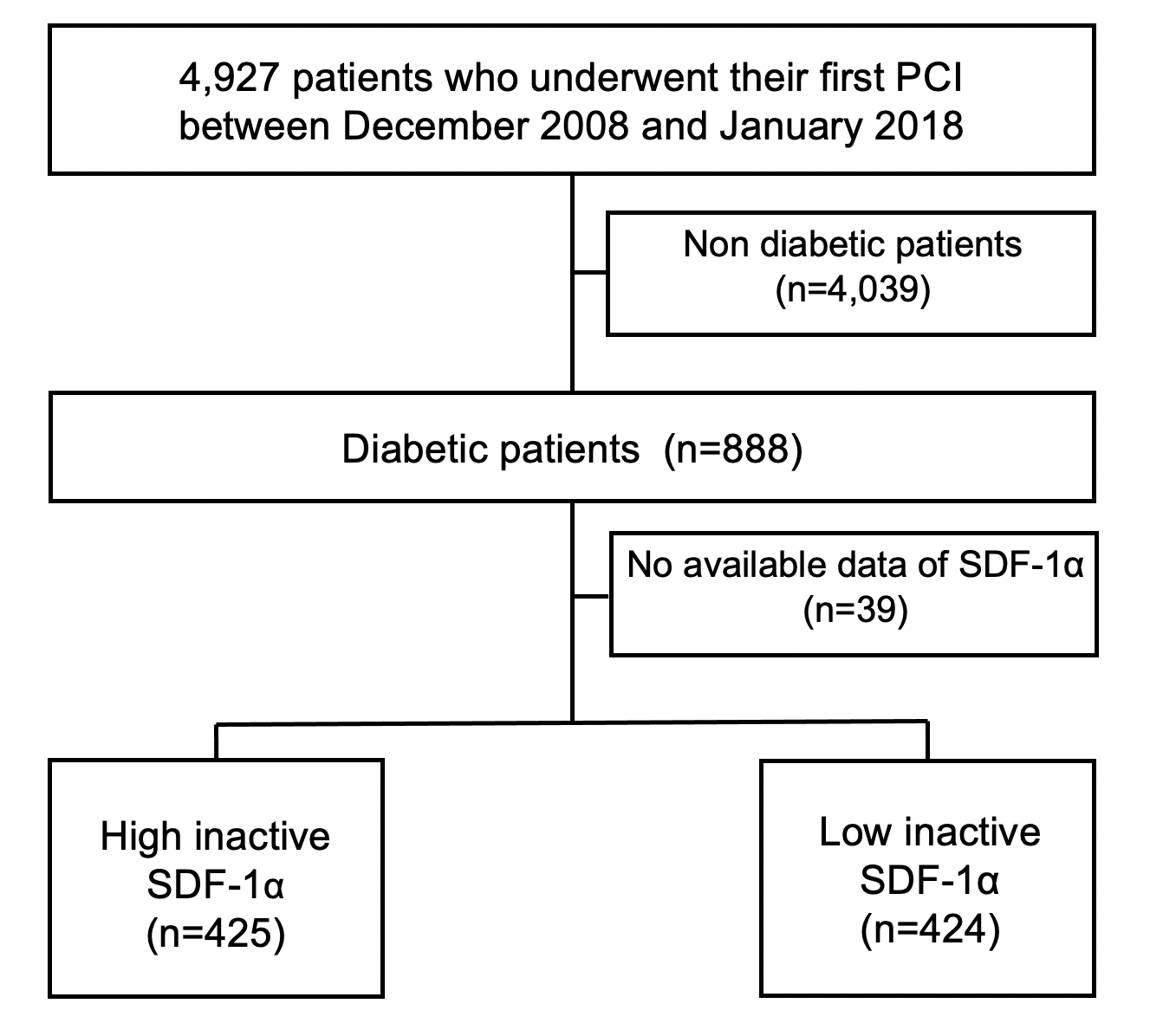

Supplement: Supplementary file 1 — Additional file 1: Table S1. Univariate Cox proportional hazard analyses for predictors of all-cause death following PCI. Table S2. Univariate Cox proportional hazard analyses for predictors of 3P-MACE following PCI. Table S3. Multivariate cox proportional hazard analyses using 3 models to assess the hazard ratios of total/active/inactive SDF-1α for all-cause death and 3P-MACE. Figure S1. Flow diagram of the study participants. [file 12933_2024_2197_MOESM1_ESM.docx]
